# Mechanistic exploration and experimental validation of the Xiaochaihu decoction for the treatment of breast cancer by network pharmacology

**DOI:** 10.18632/aging.205798

**Published:** 2024-05-13

**Authors:** Qinglong Liu, Zehua Luo, Mei Sun, Wenjun Li, Songqing Liu

**Affiliations:** 1 Department of Pharmacy, The Third Affiliated Hospital of Chongqing Medical University, Chongqing 401120, China

**Keywords:** Xiaochaihu decoction, breast cancer, network pharmacology, PI3K/Akt pathway, Q Exactive Orbitrap LC-MS/MS

## Abstract

Background: Xiaochaihu (XCH) decoction is a traditional Chinese prescription that has been recorded in the pharmacopeia of the People’s Republic of China. In China, the XCH decoction is used clinically to treat a variety of tumors, including breast cancer. However, its potential mechanism of action is still undefined.

Methods: The chemical compounds in the XCH decoction were identified via Q Exactive Orbitrap LC-MS/MS. Then, we screened the active ingredients and targets in the XCH decoction from the Traditional Chinese Medicine Systems Pharmacology Database and Analysis Platform (TCMSP). Next, Cytoscape and Metascape were used to construct an active ingredient-target-disease network, which included a protein-protein interaction (PPI) network, GO enrichment analysis, and Kyoto Encyclopedia of Genes and Genomes (KEGG) pathway analysis. Finally, we used molecular docking and *in vitro* experiments to verify the results of network pharmacology analysis.

Results: More than 70 major compounds were identified by Q Exactive Orbitrap LC-MS/MS analysis from the XCH decoction. A total of 162 active ingredients and 153 targets related to the XCH decoction and breast cancer were identified, and a compound-target-disease network was constructed. GO and KEGG analyses revealed that the XCH decoction regulated the drug response, apoptosis process, cancer pathway, and PI3K/Akt signaling pathway. Molecular docking and experimental validation indicated that the XCH decoction suppressed proliferation and induced apoptosis in breast cancer cells by regulating the expression of apoptosis-related proteins and inhibiting the PI3K/Akt pathway.

Conclusions: This study suggested that the XCH decoction can be used to treat breast cancer by inhibiting cell proliferation, inducing apoptosis and downregulating the PI3K/Akt signaling pathway.

## INTRODUCTION

Globally, breast cancer is the most common malignancy in women. According to 2019 Breast Cancer Statistics, breast cancer is the second leading cause of cancer death among American women after lung cancer [1]. Like in the United States, breast cancer is also the most common cancer among Chinese women and is the fifth leading cause of cancer death [2]. Moreover, breast cancer has become a serious disease that threatens women’s health worldwide. At present, the main treatments for breast cancer are surgery, radiotherapy, and chemotherapy. Chemotherapy is one of the most important treatments for breast cancer patients who cannot undergo surgery. However, the serious side effects caused by radiotherapy and chemotherapy often hinder the implementation of treatment plans and subsequently lead to unsatisfactory treatment results. Many studies have shown that traditional Chinese medicines (TCMs) have particular unique advantages in the treatment of cancer [3]. Studies have shown that TCM can improve the quality of life of patients with breast cancer [4]. Therefore, the study of the anti-breast cancer effect of TCM and its molecular mechanism is highly important for the treatment of breast cancer.

Xiaochaihu (XCH) decoction is a classic traditional Chinese prescription that was first described in “Shang Han Lun” (National Commission of Chinese Pharmacopoeia. It consists of seven herbs: *Bupleurum chinense* DC (Bupleuri Radix), *Scutellaria baicalensis* Georgi (Scutellariae Radix), *Codonopsis pilosula* (Franch.) Nannf. (Codonopsis Radix), *Pinellia ternata* (Thunb.) Breit. (Pinelliae Rhizoma), *Glycyrrhiza uralensis* Fisch (Gan Cao), *Zingiber officinale* Roscoe (Zingiberis Rhizoma) and *Ziziphus jujuba* Mill (Fructus Jujubae). Previous studies have shown that the XCH decoction can be used to treat a variety of diseases, including gastrointestinal diseases, respiratory diseases, and neurological disorders [5]. In addition, other studies have shown that the XCH decoction has inhibitory effects on a variety of tumors, such as lung cancer [6], colorectal cancer [7], and liver cancer [8]. In China, the XCH decoction has been used for the clinical treatment of breast cancer. Clinical observation revealed that the XCH decoction can alleviate the clinical symptoms of breast cancer patients [9]. However, the molecular mechanism by which the XCH decoction inhibits breast cancer is still unclear.

**Figure 1:**
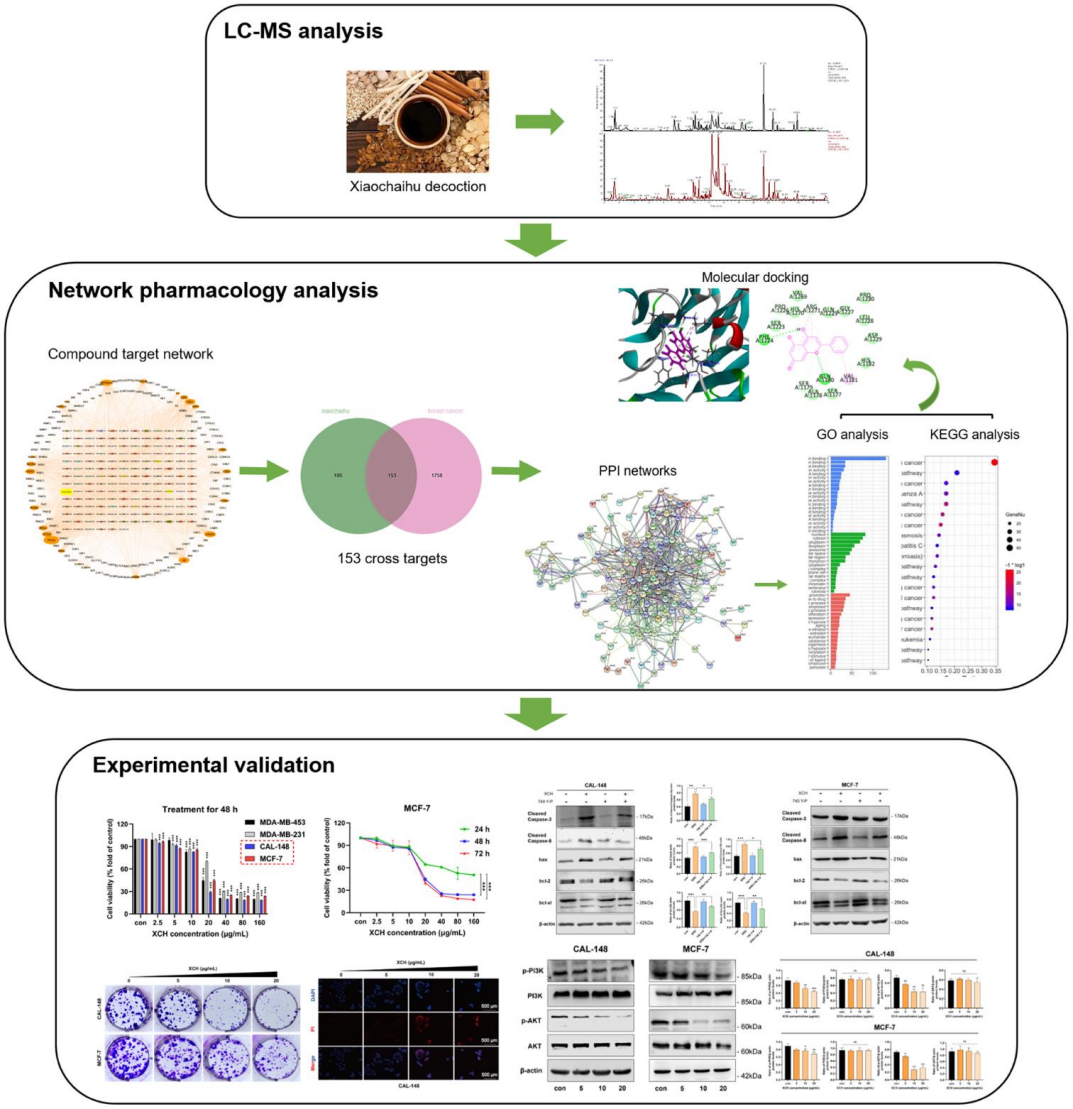
Workflow of this research.

Due to the large number of components and complex action targets of traditional Chinese medicine, conventional pharmacological approaches cannot be studied comprehensively. Therefore, we need to find new research methods. Network pharmacology is an emerging discipline that combines pharmacology, computer science, and bioinformatics [10]. Through network pharmacology, we can combine numerous traditional Chinese medicine ingredients and disease targets for comprehensive analysis. In the present study, we applied network pharmacology to explore the potential molecular mechanism of the XCH decoction in breast cancer. In addition, *in vitro* experiments were also conducted to validate the potential underlying mechanism of the XCH decoction on breast cancer, as predicted by a network pharmacology approach. Figure 1 shows the study design of this work.

## RESULTS

### Characterization of the Xiaochaihu decoction

**Figure 2:**
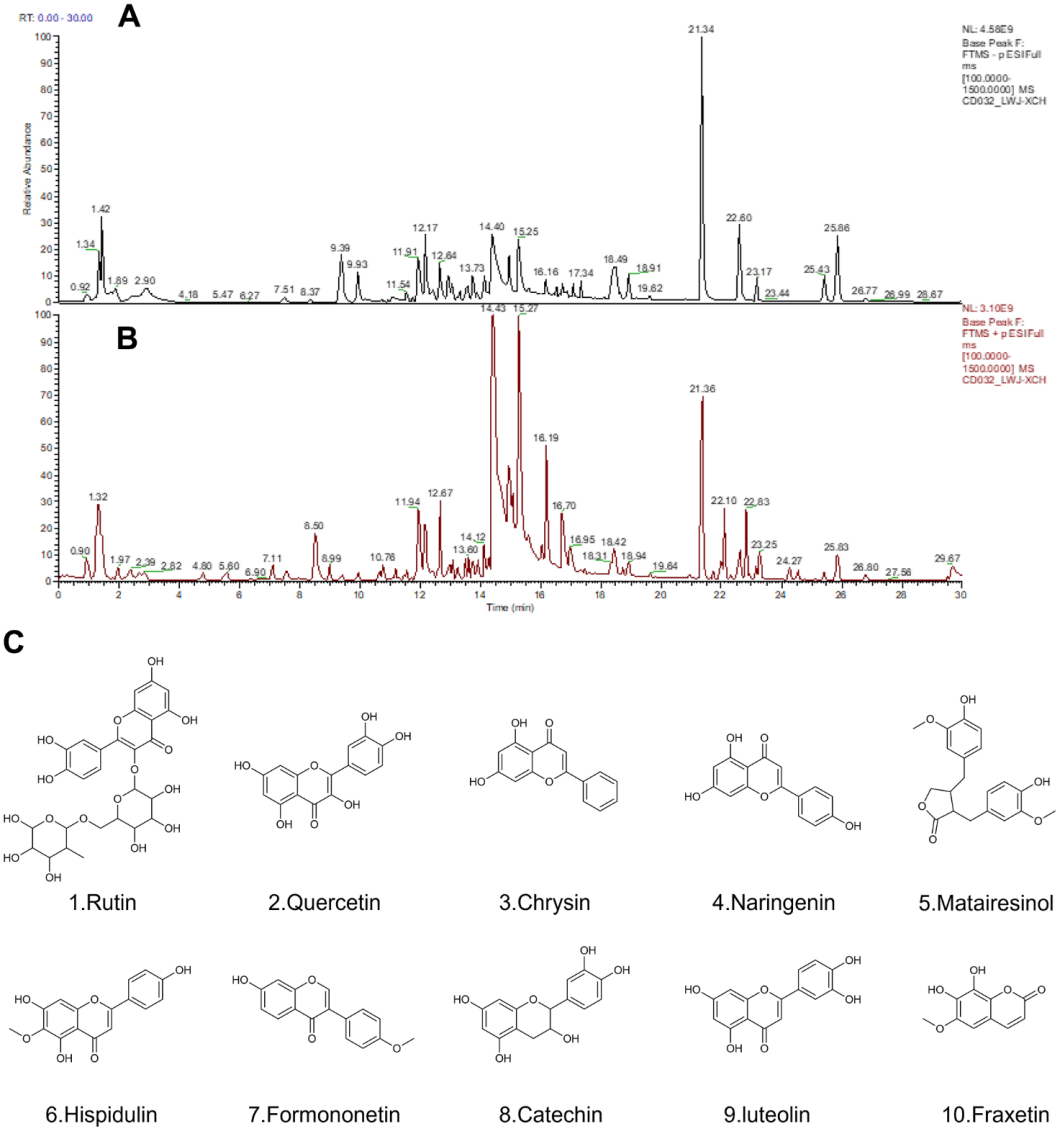
**The results of the Q Exactive Orbitrap LC-MS/MS analysis of Xiaochaihu decoction.** (**A**) Chromatography in negative-ion polarity mode; (**B**) chromatography in positive-ion polarity mode; (**C**) structures of 10 compounds obtained by Q Exactive Orbitrap LC-MS/MS analysis.

Q Exactive Orbitrap LC-MS/MS was applied for qualitative analysis of the XCH decoction. The total ion chromatograms are shown in Figure 2A, 2B under negative-ion polarity mode and positive-ion polarity mode, respectively. Over 70 principal compounds were identified from the XCH decoction using LC-MS/MS analysis. Based on the database (Thermo Fisher Scientific, Waltham, MA, USA) with a matching score greater than 80 and combined with literature reports, we screened 10 compounds related to antitumor activity, as shown in Figure 2C.

### Network of active ingredients and targets of Xiaochaihu decoction

**Figure 3:**
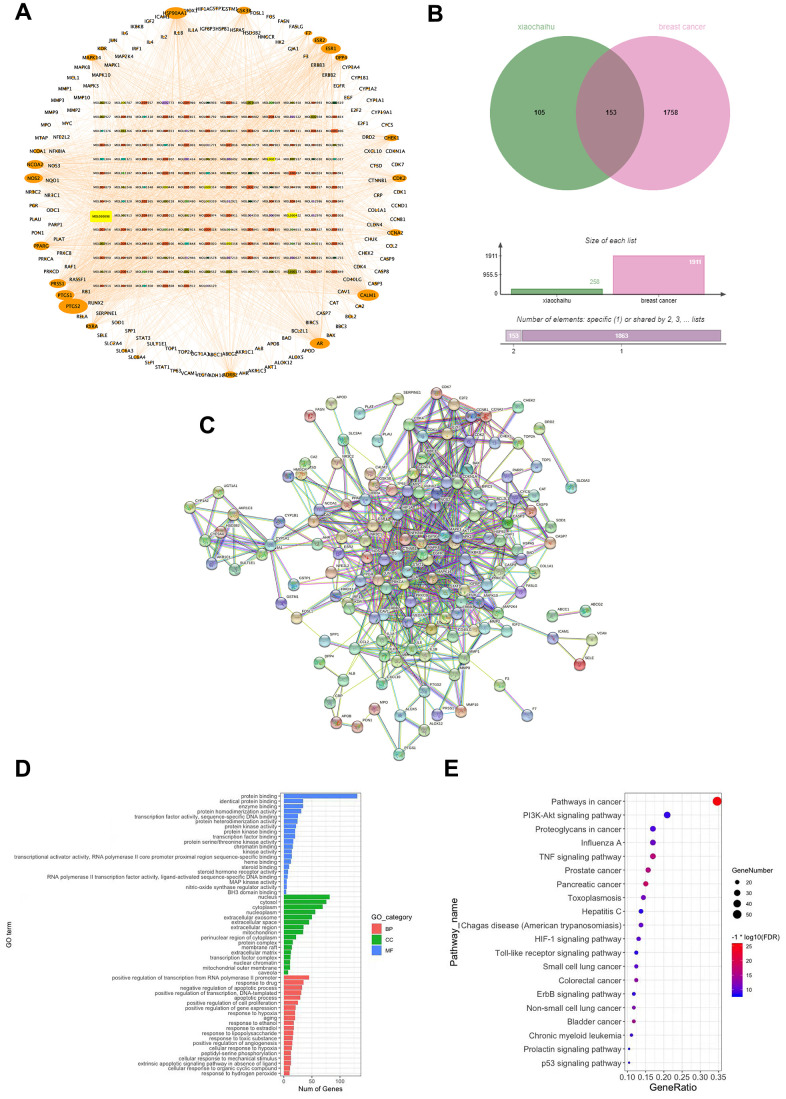
**The potential mechanisms of action of the XCH decoction against breast cancer were explored using network pharmacology.** (**A**) Active ingredient-target network of Xiaochaihu decoction; (**B**) Venn diagram of intersection targets; (**C**) Protein-protein interaction (PPI) network of Xiaochaihu decoction for breast cancer; (**D**) GO enrichment analysis; (**E**) KEGG pathway enrichment analysis.

Based on the screening criteria of bioavailability and drug-like properties, a total of 193 active ingredients of 7 traditional Chinese medicines in the XCH decoction, 163 active ingredients with corresponding targets and 258 corresponding drug targets were retrieved from the TCMSP database. An active ingredient-target network diagram of the XCH decoction was constructed using Cytoscape software, in which the outer circle graph represents the target, the inner circle graph represents the active ingredients, and the size of the graph represents the size of the node degree value (Figure 3A). There were more targets, which suggested that the effect of the active ingredient was stronger. The network graph results show a total of 421 nodes and 2300 edges. According to this network topology analysis, the most active ingredients were quercetin (DC=100), kaempferol (DC=38), wogonin (DC=33), etc., which indicated that these active ingredients may be the core active ingredients of Xiaochaihu decoction and may play a key role in its pharmacological activity.

### Xiaochaihu decoction is a potential target of the PPI network for the treatment of breast cancer

A total of 193 active ingredients, 163 active ingredients with corresponding targets and 258 corresponding drug targets of the XCH decoction were retrieved from the database. In this study, 13,970 breast cancer-related targets were retrieved from the GeneCards database. Moreover, 1,911 targets (relevance score > 10) were selected. From the intersection of drug targets and disease targets, 153 intersecting genes were obtained, and 162 of the 163 active ingredients were mapped (Supplementary Table 1), which indicated that 162 active ingredients in the XCH decoction exert therapeutic effects on breast cancer through 153 targets. PPI network analysis of the 153 targets was performed by using the String database. Figure 3C shows that the network included 153 nodes and 3223 edges, the average node degree was 42.1, and the average local clustering coefficient was 0.66. Targets with high confidence scores (combined score ≥ 0.9) were screened for mapping. These results indicate that these targets are closely related and have strong interactions.

### Bioinformatics analysis

A total of 934 items were obtained by GO and KEGG enrichment analysis. According to the condition of an FDR < 0.05, 209 items were screened, including 142 biological function (GO) items and 67 pathway (KEGG) items. There were 97 biological processes (BP) in the GO analysis, accounting for 68% of the GO enrichment entries, mainly involving drug response, negative regulation of apoptosis process, positive regulation of RNA polymerase II promoter transcription, response to estrogen, response to ethanol, etc.; molecular function (MF) mainly involved enzymes, protein, transcription factor, steroid, protein kinase and heme binding, protein homopolymerization activity, protein kinase activity, protein heterodimerization activity, transcription activation activity, etc.; and cellular component (CC) mainly involved nucleoplasm, mitochondria, membrane rafts, perinuclear region of cytoplasm, mitochondrial outer membrane, transcription factor complexes, protein complexes, extracellular matrix, etc. The top biological functions are shown in Figure 3D. KEGG pathway enrichment analysis revealed that the primary signaling pathways included cancer, PI3K/Akt, TNF, ErbB, Toll-like receptor, p53 and others (Figure 3E).

### Molecular docking to identify potential targets

**Figure 4:**
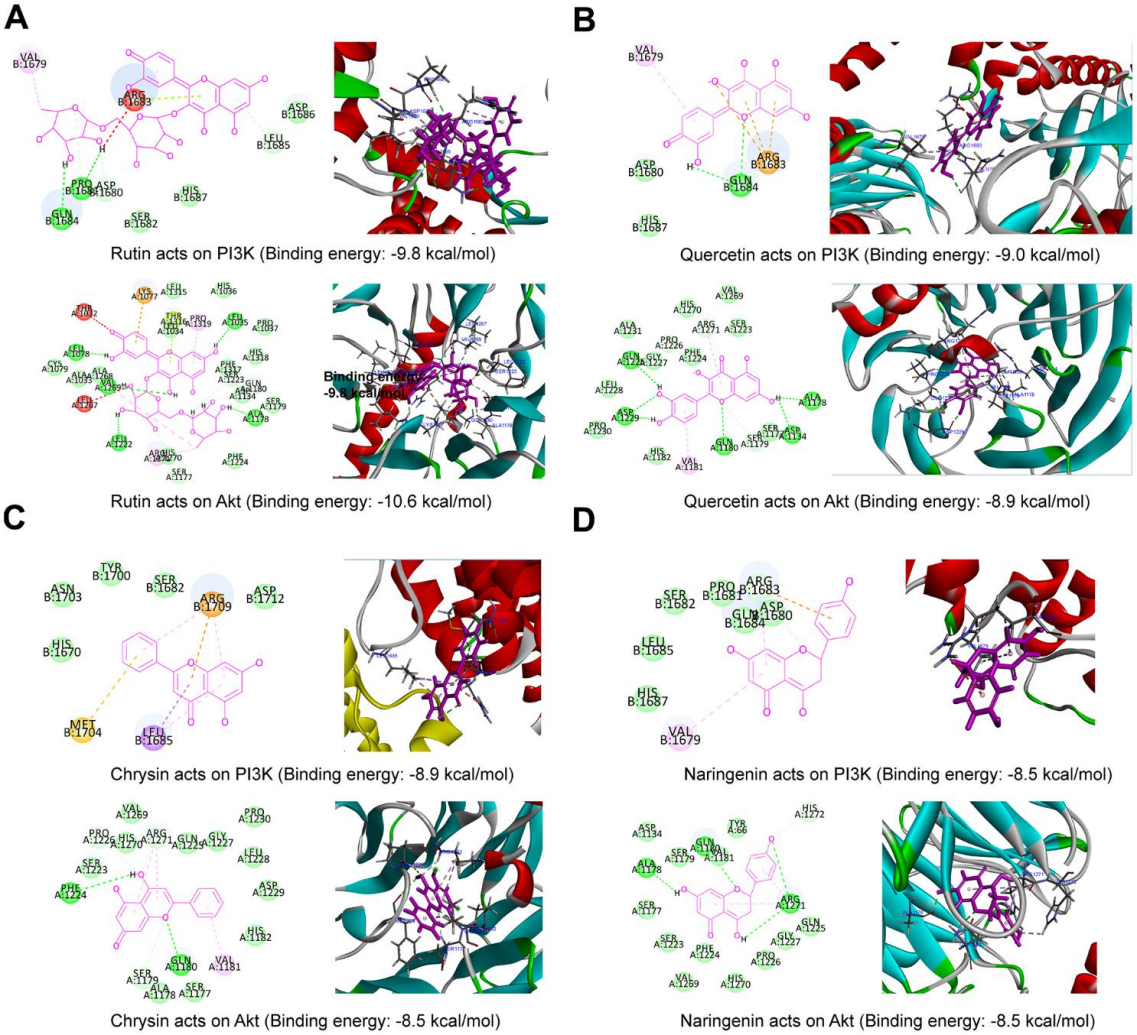
**Molecular docking result map.** (**A**) Rutin and PI3K (2D and 3D), rutin and Akt (2D and 3D); (**B**) quercetin and PI3K (2D and 3D), quercetin and Akt (2D and 3D); (**C**) chrysin and PI3K (2D and 3D), chrysin and Akt (2D and 3D); (**D**) naringenin and PI3K (2D and 3D), naringenin and Akt (2D and 3D).

Based on the PPI network analysis of the XCH decoction on breast cancer and Q Exactive Orbitrap LC-MS/MS analysis, the core active ingredients were molecularly docked with the core targets PI3K and Akt to simulate the interaction between the ingredients and targets. The binding energy was calculated by AutoDockTools 1.5.6. The greater the binding force between the protein and the compound is, the lower the binding energy. When the docking binding energy is less than -5.0 kcal/mol, the compound has a strong binding ability with the protein. As shown in Figure 4, we selected the core active ingredients (rutin, quercetin, chrysin, and naringenin) and the core targets (PI3K and Akt) for molecular docking, and the docking binding energy was less than -5.0 kcal/mol; for example, PI3K binds to rutin with a binding energy of -9.8 kcal/mol, Akt binds to quercetin with a binding energy of -8.9 kcal/mol, and Akt binds to naringenin with a binding energy of -8.5 kcal/mol. All molecular docking energy results are shown in Supplementary Table 2.

### Xiaochaihu decoction inhibits the proliferation of breast cancer cells *in vitro*

**Figure 5:**
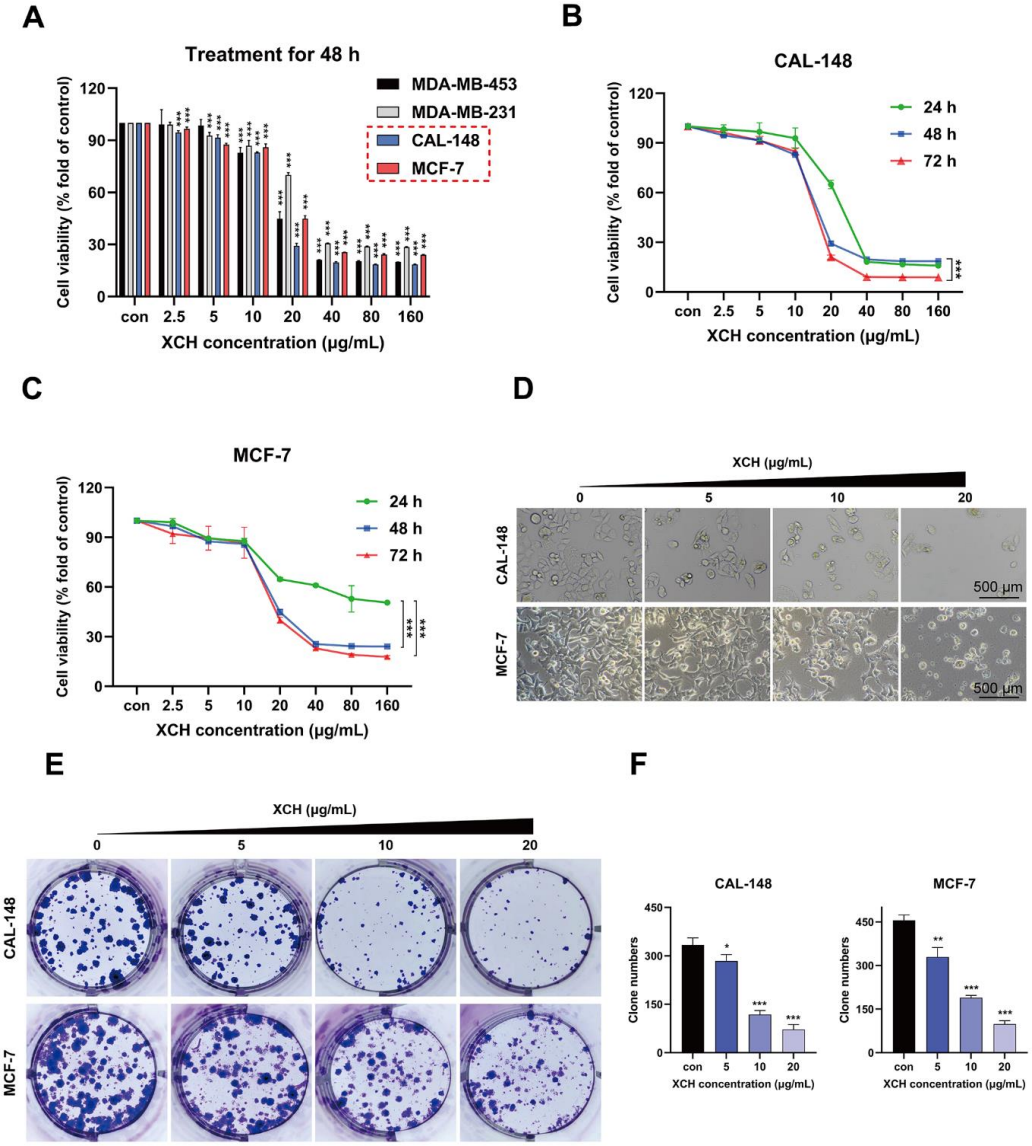
**Xiaochaihu decoction restrains breast cancer cell proliferation *in vitro*.** (**A**) The anti-breast cancer activity of Xiaochaihu decoction. The XCH decoction at various concentrations (0, 2.5, 5, 10, 20, 40, 80 or 160 μg/mL) was used to treat the four breast cancer cell lines for 48 hours. Cell viability was determined by a CCK-8 assay. (**B**, **C**) The breast cancer cell lines CAL-148 and MCF-7 were treated with different concentrations of the XCH decoction for 24, 48 or 72 hours, and viability was determined using a CCK-8 assay. (**D**) The cell state was observed under a 40x light microscope. (**E**) Effect of the XCH decoction on the colony-forming ability of the CAL-148 and MCF-7 cell lines. (**F**) Quantification of colony number. The data are shown as the mean ± SD of three experiments. *P <0.05, **P < 0.01, ***P < 0.001 compared with the control group.

To confirm the inhibitory effect of the XCH decoction on breast tumor cells, different concentrations of the XCH decoction (0, 2.5, 5, 10, 20, 40, 80 or 160 μg/mL) were used to treat different breast cancer cell lines. After 48 hours, cell viability was determined by a CCK-8 assay. The results showed that the XCH decoction inhibited the proliferation of breast cancer cells, especially CAL-148 and MCF-7 cells (Figure 5A). Subsequently, to further verify the effectiveness of the XCH decoction, CAL-148 and MCF-7 cells were treated with different concentrations of the XCH decoction for 24 h, 48 h or 72 h. The data demonstrated that the XCH decoction significantly curbed the growth of CAL-148 and MCF-7 cells in a dose- and time-dependent manner (Figure 5B, 5C). In addition, after treatment with the XCH decoction for 48 hours, cell survival decreased, as determined by 40-fold light microscopy (Figure 5D). A colony formation assay was used to evaluate the effect of the XCH decoction on proliferation, and the results showed that the number of cell clones in the XCH decoction group was dramatically less than that in the control group (Figure 5E, 5F).

### Xiaochaihu decoction induces apoptosis in breast cancer cells

**Figure 6:**
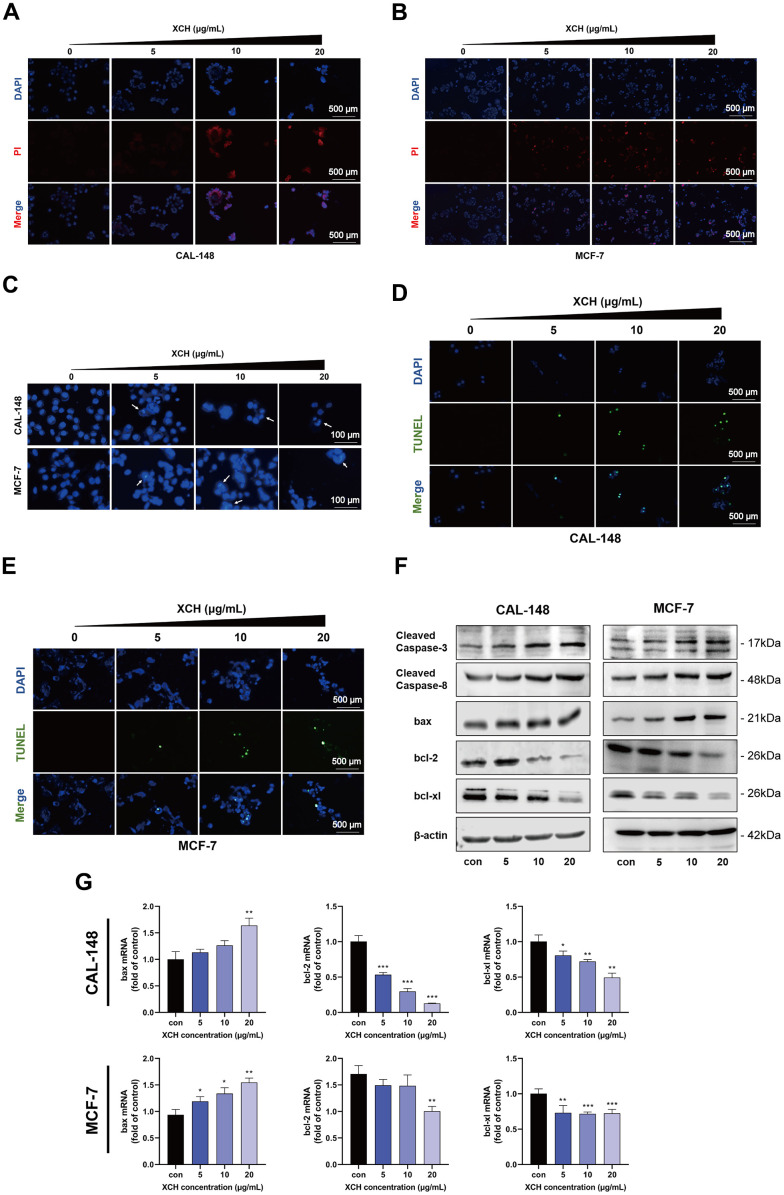
**Xiaochaihu decoction induced the apoptosis of CAL-148 and MCF-7 cells.** (**A**, **B**) Cell death was detected by propidium iodide (PI) assay. (**C**) The effects of the XCH decoction were evaluated by Hoechst 33342 analysis. (**D**, **E**) Cell apoptosis was determined by TUNEL assay. (**F**) The expression of apoptosis-related proteins was measured by Western blotting. (**G**) The mRNA levels of Bcl-2, Bcl-xl, and Bax in CAL-148 and MCF-7 cells were evaluated by RT-qPCR. The data are shown as the mean ± SD of three experiments. *P <0.05, **P < 0.01, ***P < 0.001 compared with the control group.

We evaluated the effect of the XCH decoction on cell death via a propidium iodide assay. As displayed in Figure 6A, 6B, the number of dead cells increased with increasing concentrations of the XCH decoction. Then, we used the Hoechst 33342 assay and the TUNEL assay to detect the apoptosis of breast cancer cells. With increasing concentrations of the XCH decoction, the cell brightness, degree of chromatin condensation and fragmentation increased (Figure 6C). The results of the TUNEL assay are consistent with those of the Hoechst 33342 assay. The number of cells stained with green fluorescence in the XCH decoction group was significantly greater than that in the control group (Figure 6D, 6E). Furthermore, we used flow cytometry to determine the effect of the XCH decoction on the apoptosis of CAL-148 and MCF-7 cells (Supplementary Figure 1). These results are consistent with those of the TUNEL and Hoechst 33342 assays. Subsequently, we detected the expression levels of apoptosis-related proteins in CAL-148 and MCF-7 cells by Western blotting. The results confirmed that the expression of cleaved caspase-3, cleaved caspase-8 and bax was upregulated, but the expression of bcl-2 and bcl-xl was downregulated by treatment with the XCH decoction (Figure 6F and Supplementary Figure 2). In addition, qRT-PCR was utilized to assess the expression levels of the bcl-2, bcl-xl, and bax genes. Figure 6G shows that the change in the mRNA level was concordant with the Western blot results.

### Xiaochaihu decoction induces the apoptosis of breast cancer cells by inhibiting the PI3K/Akt signaling pathway

**Figure 7:**
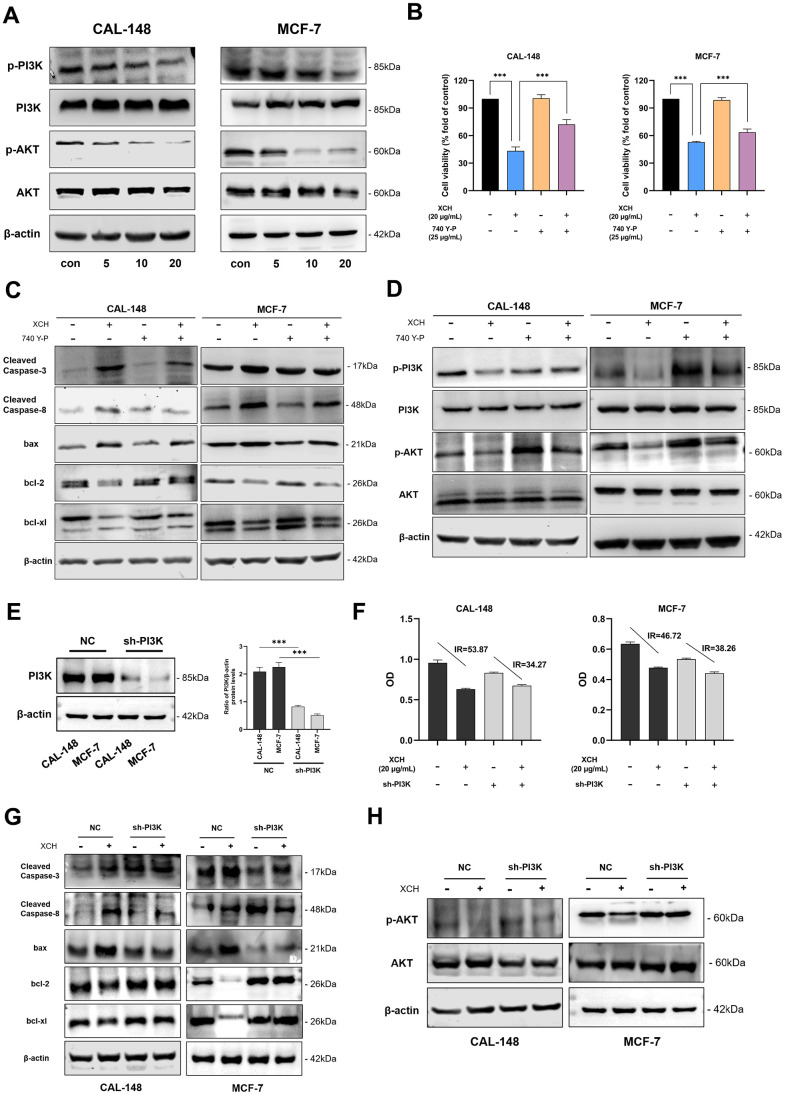
**Xiaochaihu decoction induced the apoptosis of breast cancer cells by inhibiting the PI3K/Akt signaling pathway.** (**A**) The cells were treated with the XCH decoction for 48 hours, and the protein expression of the PI3K/Akt signaling pathway was detected by Western blotting. (**B**) Cell viability was determined by CCK-8. (**C**, **D**) The cells were pretreated with a PI3K agonist (740 Y-P, 25 μg/mL) for 3 hours and then treated with or without the XCH decoction (20 μg/mL) for 48 hours. The expression levels of proteins associated with apoptosis and PI3K/Akt signaling pathway proteins were measured by Western blotting. (**E**) Knockdown of PI3K in breast cancer cells by lentivirus. (**F**) CCK-8 was used to detect the effect of the XCH decoction on breast cancer cells after PI3K knockdown. (**G**, **H**) Effect of the XCH decoction on apoptosis and PI3K pathway-related protein expression in breast cancer cells after PI3K knockdown. The data are shown as the mean ± SD of three experiments. ***P < 0.001 compared with the control group.

Previous bioinformatics analysis suggested that the anti-breast cancer mechanism of the XCH decoction may involve regulating the PI3K/Akt pathway. To verify the results of the bioinformatics analysis, PI3K/Akt pathway-related proteins were further detected by Western blot. The levels of phosphorylated PI3K and phosphorylated Akt were markedly decreased after treatment with the XCH decoction (Figure 7A). To confirm the results of the experiment, we analyzed cell viability after treatment with the XCH decoction with or without a PI3K agonist (740Y-P). As shown in Figure 7B, the XCH decoction obviously inhibited cell viability. However, the PI3K agonist attenuated the XCH decoction-induced decrease in cell viability. Next, we measured the expression levels of proteins associated with apoptosis and the PI3K/Akt signaling pathway. Similar to the results of the CCK-8 assay, the PI3K agonist reversed the effects of the XCH decoction on breast cancer cells and the inhibition of the PI3K/Akt pathway (Figure 7C, 7D and Supplementary Figure 3). In addition, we constructed PI3K-knockdown CAL-148 and MCF-7 cells to verify the above experimental results (Figure 7E). The results showed that the inhibition of breast cancer cells by the XCH decoction was attenuated when PI3K was knocked down (Figure 7F). Moreover, compared with those in the NC group, the effects of XCH on the expression of apoptosis- and PI3K pathway-related proteins in the PI3K knockdown group were partially attenuated (Figure 7G, 7H and Supplementary Figure 4). Collectively, these results suggest that the anti-breast cancer mechanism of the XCH decoction might be associated with the regulation of the PI3K/Akt pathway.

## DISCUSSION

Surgery combined with chemoradiotherapy is the most common therapy for clinical breast cancer. Nevertheless, surgery for advanced or metastatic breast cancer does not improve prognosis, and long-term chemoradiotherapy can cause severe side effects, such as bone marrow suppression, radiation pneumonia, and radiation dermatitis [11, 12]. The XCH decoction is a traditional Chinese prescription. Because of its good anti-inflammatory effect, it is commonly used in China to treat chronic hepatitis, acute pancreatitis, gastroesophageal reflux disease and other diseases [13–15]. In addition, some clinical studies have shown that the XCH decoction can inhibit breast cancer. However, there is a lack of research on the molecular mechanism by which the XCH decoction affects breast cancer. In our study, the possible mechanism of the XCH decoction for treating breast cancer was investigated by network pharmacology, and breast cancer cell lines were used to evaluate the anti-breast cancer effect of the XCH decoction and verify the results of network pharmacological analysis.

The XCH decoction contains seven herbs, including Bupleuri Radix, Scutellariae Radix, Codonopsis Radix, Pinelliae Rhizoma, Glycyrrhizae Radix et Rhizoma, Zingiberis Rhizoma and Fructus Jujubae. In this study, Q Exactive Orbitrap LC-MS/MS was used to analyze the chemical composition of the XCH decoction. More than 70 major compounds, mostly flavonoids, were identified. Subsequently, network pharmacology was used to explore the potential mechanism of the XCH decoction against breast cancer. Combined with a compound-target-pathway network and Q Exactive Orbitrap LC-MS/MS analysis, we found that rutin, quercetin, chrysin, and naringenin, which are present at relatively high concentrations in the XCH decoction, might be the most effective compounds for treating breast cancer. Rutin, also known as rutoside, is a flavonoid glycoside found in many fruits and vegetables. Numerous studies have demonstrated the anticancer effects of rutin [16]. Recent findings have shown that rutin can inhibit tumor cell proliferation, migration and invasion and induce tumor cell apoptosis [17]. Elsayed et al. reported that rutin suppressed breast cancer cells via the abolition of c-met/HGF signaling and its downstream cascades [18]. Quercetin is a natural flavonoid with a variety of biological activities. Many studies have shown that quercetin has good anti-breast cancer effects [19, 20]. Quercetin markedly inhibited breast cancer cell proliferation by influencing the G1 phase by downregulating the expression of cyclin D1 and P21 [21]. Some studies have also shown that quercetin induces apoptosis and increases the expression of proapoptotic proteins such as bax and bak; alternatively, it decreases the expression of the antiapoptotic protein bcl-2 in the CAL-148 and MCF-7 cell lines [22]. Chen et al. reported that quercetin inhibits the migration and invasion of tumors by regulating the expression of epithelial–mesenchymal transition (EMT)-related proteins [23]. The Notch1 and PI3K/Akt signaling pathways were suppressed in breast cancer stem cells after quercetin treatment [24]. Additionally, quercetin resensitizes docetaxel-resistant breast cancer cells by inhibiting the expression of Lef1 and decreasing Smad-dependent TGF-β signaling pathway activation [25]. Chrysin is also a flavonoid that is widespread in many plant extracts, honey, and propolis [26]. Chrysin generally blocks various tumor cell lines [27]. Experimental studies have shown that chrysin can inhibit tumor progression by regulating the H19/let-7a/COPB2, P38-MAPK/Akt and Akt/mTOR pathways and affecting mitochondrial function and ER stress [28–32]. Naringenin is a major citrus flavonoid found primarily in grapes and oranges. Naringenin has numerous biological effects, such as anticancer, anti-inflammatory, and antiviral effects [33]. In a breast cancer study, the experimental results revealed that naringenin can induce apoptosis and reduce autophagy in breast cancer cells [34, 35]. Additionally, naringenin could enhance the sensitivity of breast cancer to chemotherapeutic drugs [36, 37]. Furthermore, the potential molecular mechanism and signaling pathway involved in the effects of the XCH decoction were predicted by GO and KEGG enrichment analyses. The results showed that the enriched biological processes in the XCH decoction included cell proliferation, apoptotic processes, and response to toxic substances. Pathway enrichment analysis revealed that the cancer pathway, TNF signaling pathway, HIF-1 signaling pathway, PI3K/Akt signaling pathway, and Tp53 signaling pathway were enriched in the XCH decoction group. In particular, the PI3K/Akt pathway was among the top three pathways according to KEGG enrichment analysis (Figure 3E). The PI3K/Akt signaling pathway is abnormally overexpressed in tumors and plays a key regulatory role in tumor progression [38]. In breast cancer, the PI3K/Akt pathway is frequently deregulated by multiple mechanisms, leading to dysfunction of PI3K/Akt signaling. Therefore, regulating the PI3K pathway may be an effective treatment for breast cancer. Various studies have revealed that inhibiting the PI3K pathway effectively blocks proliferation, invasion, and migration and induces death in breast cancer cells [39].

Next, we further verified the network pharmacology prediction results via *in vitro* experiments. The XCH decoction drastically inhibited proliferation and induced apoptosis in multiple breast cancer cell lines in a dose- and time-dependent manner. Furthermore, we examined the expression of proteins related to cell apoptosis and the PI3K/Akt signaling pathway. In addition, we used molecular docking to detect the binding of active compounds based on Q Exactive Orbitrap LC-MS/MS and network pharmacology to PI3K and Akt. These results were similar to those predicted by network pharmacological analysis. However, we acknowledge that the present study has several limitations. For example, a nude mouse xenograft model was not established in this study to evaluate the anti-breast cancer effect of the XCH decoction *in vivo*. In addition, in this study, only the TCMSP database was used to analyze and predict the active ingredients and targets of the XCH decoction, and other databases were not included, which may have led to the loss of some information. Collectively, our findings show that the XCH decoction inhibits breast cancer in a variety of ways through its active compounds and targets. The XCH decoction can suppress breast cancer cell proliferation and induce apoptosis by downregulating the PI3K/Akt pathway.

## CONCLUSIONS

In summary, the current experimental data show that the XCH decoction significantly inhibits breast cancer cell proliferation and induces apoptosis *in vitro*. Network pharmacological analysis suggested that the XCH decoction may exert its anti-breast cancer effects through regulating the PI3K/Akt signaling pathway. Moreover, we validated the results of the network pharmacology analysis using PI3K agonists and constructed PI3K-knockdown breast cancer cells. In conclusion, our study preliminarily revealed the mechanism underlying the anti-breast cancer effect of the XCH decoction and provided a theoretical basis for the clinical application of the XCH decoction in the treatment of breast cancer.

## MATERIALS AND METHODS

### Reagents

Dulbecco’s modified Eagle’s medium (DMEM) and fetal bovine serum (FBS) were obtained from Biological Industries (Israel). Then, 1% penicillin/streptomycin, 4% paraformaldehyde, crystal violet staining solution, Hoechst 33342, RIPA buffer, and an Enhanced BCA Protein Assay Kit were acquired from Beyotime Biotechnology (Shanghai, China). The Cell Counting Kit-8 (CCK-8) assay, 2 × SYBR Green qPCR Master Mix, protease inhibitor, phosphatase inhibitor and anti-β-actin were purchased from Bimake (Houston, TX, USA). A total RNA extraction kit was obtained from TIANGEN Biotech (Beijing, China). MonScript™ RTIII Super Mix with dsDNase (two steps) was obtained from Monad (Wuhan, China). A propidium iodide test kit was purchased from Dalian Meilun Biotechnology (Dalian, China). A TUNEL apoptosis assay kit was obtained from Vazyme (Nanjing, China). Primary antibodies against Akt, phospho-Akt, Bax, Bcl-xl and Bcl-2 were obtained from ProteinTech Group, Inc. (Chicago, IL, USA). The following primary antibodies were procured from Affinity Biosciences (Zhenjiang, China): anti-PI3K, anti-phospho-PI3K, anti-cleaved Caspase-3 and anti-cleaved Caspase-8. The secondary antibody (IRDye® 800CW goat anti-rabbit IgG (H&L)) was purchased from Abcam (Cambridge, United Kingdom). 740 Y-P were obtained from MedChemExpress (NJ, USA). An Annexin V-FITC/PI Apoptosis Assay Kit was obtained from Biosharp (Hefei, China).

### Xiaochaihu (XCH) decoction preparation

The XCH decoction (Table 1) was prepared according to these steps: first, all Chinese herbal medicines (Bupleuri Radix 20 g, Scutellariae Radix 10 g, Codonopsis Radix 10 g, Pinelliae Rhizoma 10 g, Glycyrrhizae Radix et Rhizoma 5 g, Zingiberis Rhizoma 5 g, Fructus Jujubae 6 g) were crushed and mixed, and then 200 mL of distilled water was added for 3 hours. After being filtered and concentrated to 1 g/mL, the combined decoction was kept at -20° C until use.

**Table 1:** The compound composition of Xiaochaihu decoction.

**Herb**	**Binomial nomenclature**	**Weight (g)**
Chai Hu	Radix Bupleuri	20
Huang Qin	Radix Scutellariae baicalensis	10
Dang Shen	Codonopsis pilosula	10
Ban Xia	Rhizoma Pinelliae Tematae	10
Gan Cao	Radix Glycyrhizae Uralensis	5
Gan Jiang	Rhizoma Zingiberis	5
Da Zao	Fruc-tus Zizyphi Jujubae	6
Total		66

### Q Exactive Orbitrap LC-MS/MS analysis

An InertSustain AQ-C18 (2.1×150 mm, i.d.; 1.8 μm, Shimadzu, Japan) was used to separate the samples at 35° C. Methanol and 0.1% formic acid-water (95:5) were applied as the mobile phase. The flow rate and injection volume were 0.3 mL/min and 5 μL, respectively. Nitrogen was used as the auxiliary gas. The mass was determined based on positive and negative scanning modes with a m/z of 100-1500.

### Screening of active ingredients and related targets in Xiaochaihu decoction and breast cancer

The effective components of 7 kinds of traditional Chinese medicine (TCM) in Xiaochaihu decoction were searched in turn by using TCMSP (https://www.tcmsp-e.com/#/database; traditional Chinese Medicine Systems Pharmacology Database and Analysis Platform). Subsequently, an oral bioavailability (OB) greater than 30% and a drug likeness (DL) greater than 0.18 were used as screening criteria, and the active ingredients of 7 kinds of TCM were screened according to the conditions. Based on the search function of related targets of TCMSP, the target genes corresponding to the active ingredients, namely, drug target genes, were searched. The Perl package (ActivePerl-5.28) was used to convert these target genes into gene symbols for subsequent research. Then, with “breast cancer” as the keyword, genes related to breast cancer, which were called disease target genes, were searched in the GeneCards database (https://www.genecards.org/). After deleting duplicate data, these target genes were normalized in the UniProt database.

### Construction of the active ingredient-target network

To investigate the pharmacological mechanisms of the Xiaochaihu decoction and further observe the biological functions of the active ingredients and possible targets, Cytoscape software was used to construct a composite target network for 162 potential active ingredients and their related targets of the Xiaochaihu decoction. The potential targets of Xiaochaihu decoction were subsequently uploaded to the Metascape database for pathway and process enrichment analysis [40]. The data were screened and collected when the p-value was less than 0.01, the enrichment factor was greater than 1.5, and the minimum count was 3. Based on the kappa-values of their gene associations, the significant terms were clustered into a tree, and the tree was then cast into term clusters by applying the 0.3 kappa-value threshold.

### Protein–protein interaction (PPI) network construction

To identify potential targets of the Xiaochaihu decoction in breast cancer treatment, Perl was used to intersect the drug targets and disease targets obtained as described in Section 1.1. A Venn diagram of the intersection of common targets of Xiaochaihu decoction and breast cancer was generated. Then, these data were imported into the STRING database (http://string-db.org/) with a combined score greater than 0.9 and the species set to “*Homo sapiens*”. Targets were selected according to these conditions for further enrichment analysis, and finally, the PPI was obtained.

### Biological function and pathway enrichment analysis

Initially, the common targets of the Xiaochaihu Decoction and breast cancer were imported into the DAVID database (https://david.ncifcrf.gov/, Version 6.8). Gene Ontology (GO) enrichment analysis was performed for three categories: biological process (BP), molecular function (MF) and cellular component (CC). Kyoto Encyclopedia of Genes and Genomes (KEGG) pathway analysis was subsequently performed. We set an FDR < 0.05 as the threshold and screened the significantly enriched GO and KEGG terms. The R language (3.6.0) was used to visualize the top-ranked entries.

### Molecular docking

The core active ingredients screened based on the above methods and LC-MS/MS will be stored in the PubChem database (https://pubchem.ncbi.nlm.nih.gov/). The corresponding small molecule ligand structures were downloaded and saved for future use. Subsequently, the screened core disease-related targets were entered into the PDB database (https://www.rcsb.org/), after which the 3D structures of the corresponding gene proteins were downloaded and saved. The LibDock model of Discovery Studio 2019 was used for molecular docking.

### Cell culture

The human breast cancer cell lines CAL-148, MCF-7, MDA-MB-231, and MDA-MB-453, which were cultured in DMEM supplemented with 10% FBS and 1% penicillin/streptomycin and incubated with 5% CO_2_ at 37° C, were obtained from the Chinese Academy of Sciences (Shanghai, China).

### Cell viability assay

The effects of the XCH decoction on cell viability were determined by the CCK-8 assay. Human breast cancer cell lines were plated in 96-well plates (3×10^3^/well). The next day, fresh DMEM supplemented with 0-160 μg/mL XCH decoction was added, and the cells were incubated for 24-72 h. Afterward, 10 μL of CCK-8 solution was added to each well, which was mixed with serum-free medium in an incubator at 37° C for 2 hours. Afterward, the absorbance was determined with a SynergyH1 microplate spectrophotometer (Bio Tek) at 450 nm. To assess cell morphology, CAL-148 and MCF-7 cells were examined under a light microscope at 40× magnification (Nikon, Japan).

### Colony formation assay

CAL-148 and MCF-7 cells were seeded in 24-well plates (approximately 500 cells per well). On the following day, the medium was changed to fresh medium supplemented with various concentrations (0, 5, 10 or 20 μg/mL) of the XCH decoction, and the medium was replaced every 3 days without passing through the cells for a period of 12 days. Thereafter, the cells were fixed for 20 min in 4% paraformaldehyde and subsequently stained for 20 min with crystal violet staining solution. Colony numbers were subsequently recorded using a digital camera and quantitated with ImageJ software.

### Quantitative real-time PCR (qRT-PCR)

A Total RNA Extraction Kit was used to extract total RNA from CAL-148 and MCF-7 cells. Afterward, a total RNA sample was reverse-transcribed into cDNA following the manufacturer’s instructions for the MonScript™ RTIII Super Mix with dsDNase (two steps). Ultimately, for measuring the expression level of mRNA, qRT-PCR was performed using 2 × SYBR Green qPCR Master Mix in QuantStudio Dx (Applied Biosystems, CA, USA). The primer pairs used are listed in Table 2. β-Actin was used as a control gene, and the amount of RNA was computed using the 2-ΔΔCt method.

**Table 2:** Primer sequences used for the qPCR analysis.

**Gene**	**Forward primer, 5’-3’**	**Reverse primer, 5’-3’**
bax	ACATGGAGCTGCAGAGGATG	GACAGGGACATCAGTCGCTT
bcl-xl	GCCATCAATGGCAACCCATC	TCCACAAAAGTATCCTGTTCAAAGC
bcl-2	GGTGGGGTCATGTGTGTGG	CGGTTCAGGTACTCAGTCATCC
β-actin	AGGGTGTTGTGGAGATGGG	TGGCCTTGAGTTTCCTGCT

### Propidium iodide (PI) assay

A PI assay was used to measure the degree of breast cancer cell death induced by the XCH decoction. CAL-148 and MCF-7 cells were seeded in 24-well plates (1 × 10^4^ cells/well). After cell attachment and removal of the old medium, the cells were incubated in fresh complete culture medium containing various concentrations (0, 5, 10 or 20 μg/mL) of the XCH decoction for 48 hours. Then, a 1× PBS solution was used to wash the cells twice after removal of the cell culture medium. The cells were stained with a propidium iodide test kit and photographed by fluorescence microscopy at 100× magnification (Nikon).

### Hoechst 33342 analysis

Hoechst 33342, a cell permeable nuclear dye, was used to stain the cells for apoptosis analysis. The CAL-148 and MCF-7 cell lines were cultured in 24-well plates (1 × 10^4^ cells per well). The next day, cells treated with different concentrations (0, 5, 10 or 20 μg/mL) of the XCH decoction were cultivated at 37° C for 48 h. Afterward, the cells were stained with Hoechst 33342 for 10 min in an incubator and then washed twice with PBS before being evaluated under a fluorescence microscope at 200× magnification (Nikon).

### TUNEL assay

Apoptosis was determined using the TUNEL assay. CAL-148 and MCF-7 cells were added to 24-well plates (1 × 10^4^ cells/well) and incubated overnight. Fresh medium containing different concentrations of the XCH decoction was added to the wells and incubated for 48 h. The TUNEL assay was performed according to the manufacturer’s instructions. Fluorescence signals were captured using fluorescence microscopy at 200× magnification (Nikon).

### Flow cytometry analysis

CAL-148 and MCF-7 breast cancer cells were plated in 6 cm dishes and then treated with different concentrations (0, 5, 10, or 20 μg/mL) of the XCH decoction. After 48 h, the cells were collected and washed with cold PBS and suspended in 250 μL of 1× binding buffer, followed by the addition of 5 μL of FITC Annexin V and 10 μL of PI and incubation for 10 min at room temperature in the dark. Finally, after the addition of 400 μL of 1× binding buffer, the samples were analyzed with a CytoFLEX flow cytometer (Beckman Coulter).

### Construction of PI3K-knockdown breast cancer cells

The PI3K shRNA plasmid was procured from MIAOLING BIOLOGY (Hubei, China), with the PI3K shRNA target sequence 5’- GCATTAGAATTTACAGCAAGA-3’. The PI3K shRNA plasmid and NC shRNA plasmid were transfected into HEK 293T cells using Lipo8000™ Transfection Reagent (Beyotime, China) to generate viral particles. After 48 hours of transfection, the virus particles in the cell culture supernatant were filtered through a 0.45 μm membrane and used for infection of CAL-148 and MCF-7 cells at 37° C in the presence of a HiTransG P Virus Infection Enhancer (GENECHEM, China). Puromycin (1 μg/mL) was added for the selection of stably infected cells. The knockdown efficiency was confirmed through Western blot analysis.

### Western blotting

Total protein was extracted from the cells with cold RIPA buffer containing 1 mM protease inhibitor and 1 mM phosphatase inhibitor. After sonication and centrifugation, the supernatant of the protein lysate was analyzed with an Enhanced BCA Protein Assay Kit. Subsequently, thirty micrograms (30 μg) of denatured proteins were separated by 8-12% sodium dodecyl sulfate-polyacrylamide gel electrophoresis and then electrotransferred to polyvinylidene fluoride (PVDF) membranes (Millipore, Billerica, MA, USA). After soaking in rapid blocking solution for 40 min, the membranes were incubated overnight with primary antibodies at 4° C. Later, the protein bands were incubated with fluorescent secondary antibodies for 2 hours at room temperature. Afterward, the bands were detected with an Odyssey® CLx Imaging System (LI-COR Biosciences, USA). Eventually, the relative expression levels of the proteins were determined with ImageJ software and analyzed with GraphPad Prism 8.0.2 software.

### Statistical analysis

In this study, all experiments were repeated at least three independent times, and the quantitative experimental data are reported as the means ± SDs. GraphPad Prism 8.0.2 was used for the statistical analysis. Our statistical analysis was based on one-way ANOVA and Tukey’s multiple comparison test. Statistical significance was defined as a P-value less than 0.05.

### Data availability

All data supporting the findings are available within the article and its Supplementary Information; they can be requested on reasonable grounds.

## Supplementary Material

Supplementary Figures

Supplementary Table 1

Supplementary Table 2
