# Supplementary material for: Mechanistic exploration and experimental validation of the Xiaochaihu decoction for the treatment of breast cancer by network pharmacology
Source: Aging (Albany NY). 2024 May 13;16(9):7979–99. doi: 10.18632/aging.205798 (PMC11132012; doi:10.18632/aging.205798)
Supplement: Supplementary Figures [file aging-16-205798-s001.pdf]

SUPPLEMENTARY FIGURES

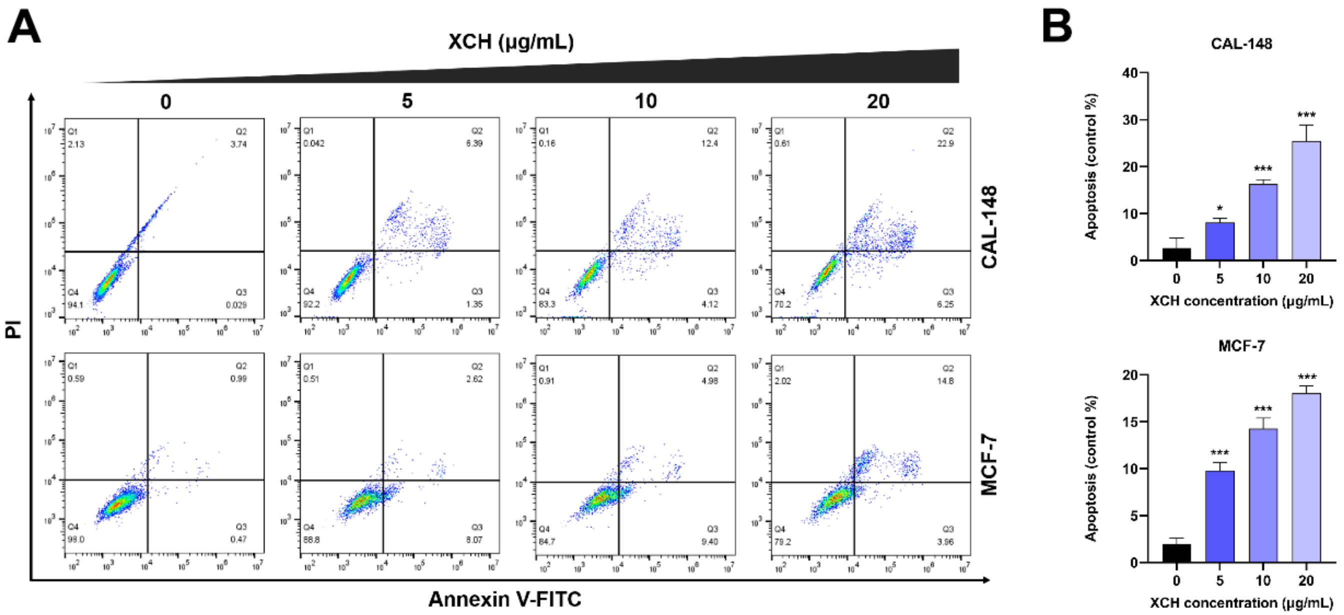

Supplementary Figure 1. (**A**, **B**) Detection of the effect of XCH decoction on breast cancer cell apoptosis by flow cytometry. The data were shown as the mean ± SD of three experiments. **P* <0.05, ***P* < 0.01, ****P* < 0.001 compared with the control group.

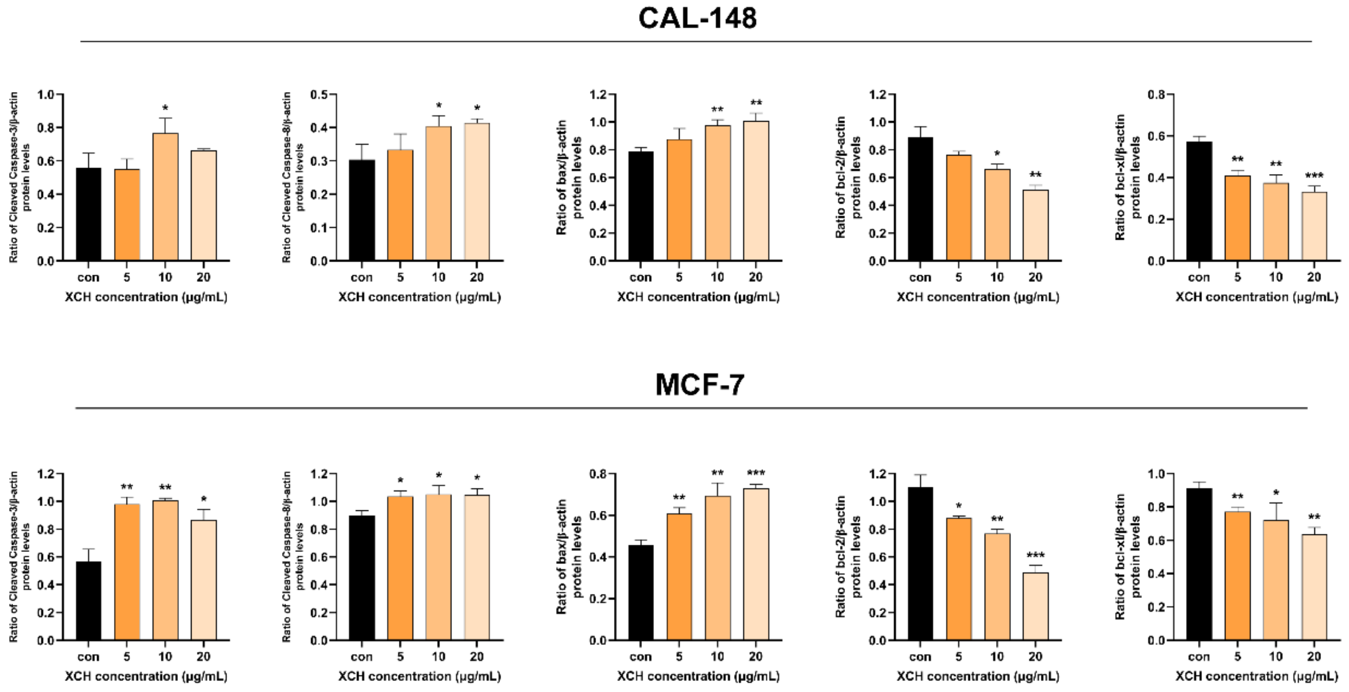

Supplementary Figure 2. Quantification of Cleaved caspase 3, 8, bax, bcl-2 and bcl-xl. The data were shown as the mean ± SD of three experiments. **P* <0.05, ***P* < 0.01, ****P* < 0.001 compared with the control group.

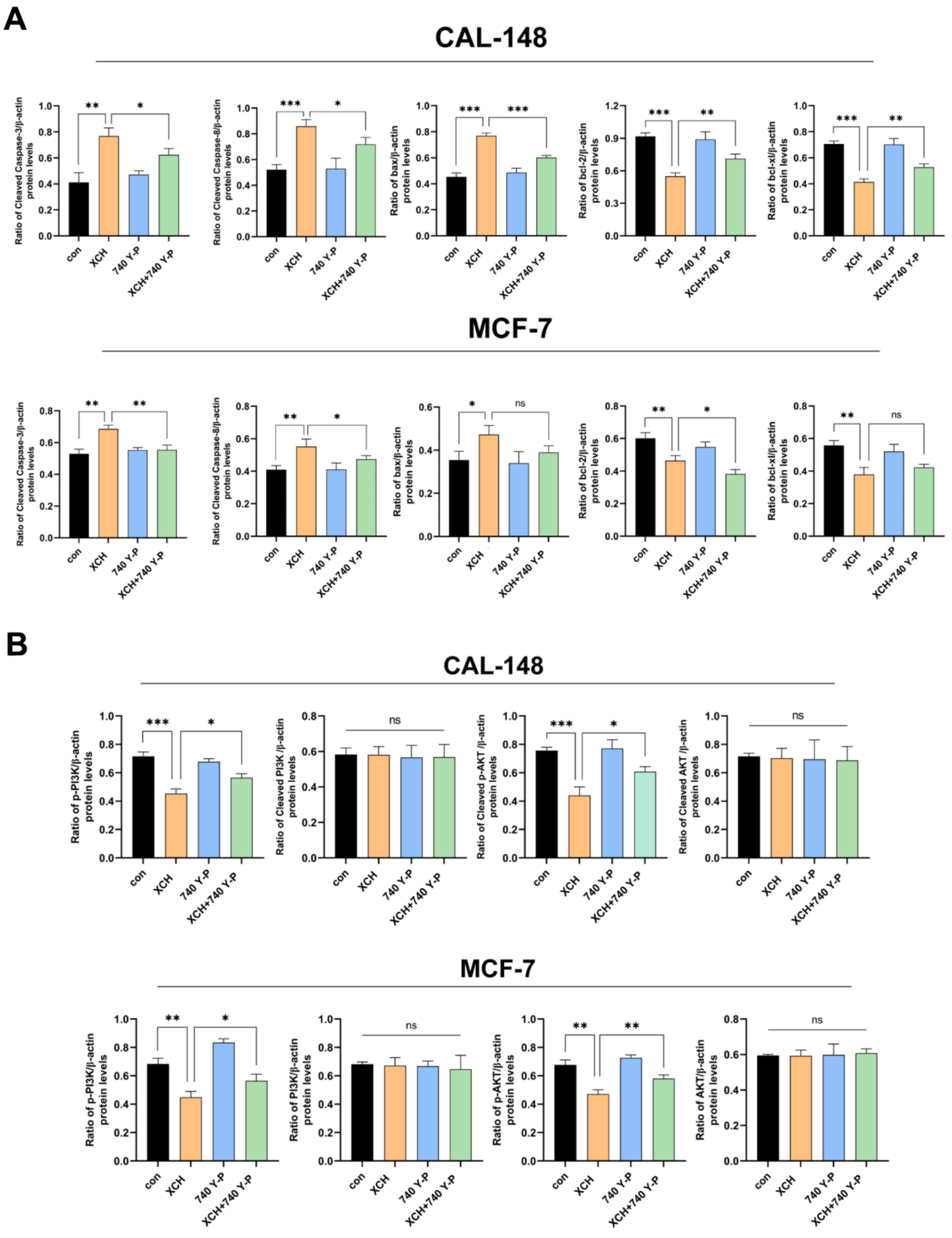

Supplementary Figure 3. (**A**, **B**) Quantification of Western blot. Cells were treated with or without XCH decoction for 48 h after PI3K agonist (740 Y-P, 25 μg/mL) pretreatment for 2 h. The data were shown as the mean ± SD of three experiments. **P* <0.05, ***P* < 0.01, ****P* < 0.001 compared with the control group.

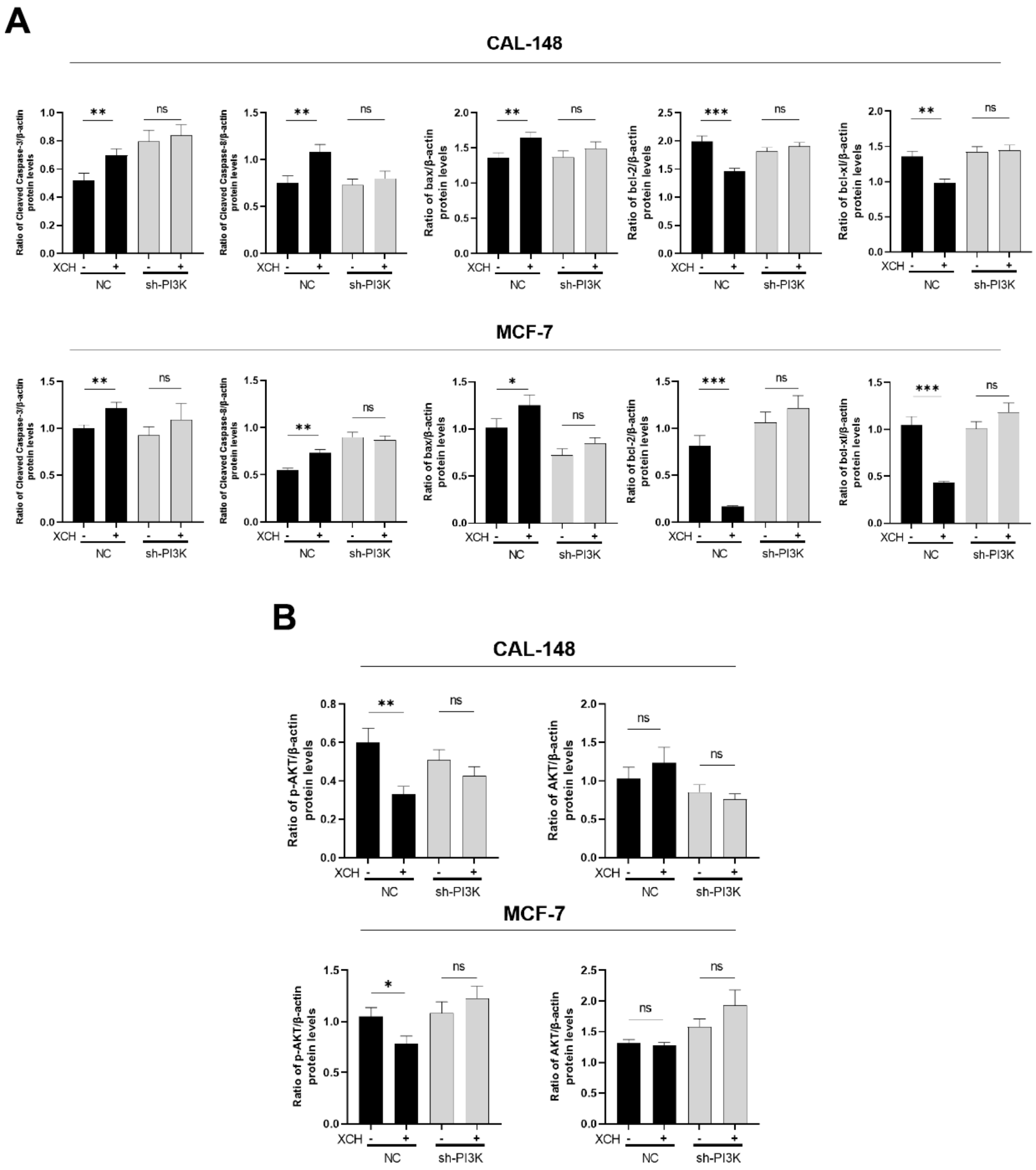

Supplementary Figure 4. (**A**, **B**) Quantification of Western blot. The data were shown as the mean ± SD of three experiments. **P* <0.05, ***P* < 0.01 compared with the control group.
