# Supplementary material for: Mechanistic exploration and experimental validation of the Xiaochaihu decoction for the treatment of breast cancer by network pharmacology
Source: Aging (Albany NY). 2024 May 13;16(9):7979–99. doi: 10.18632/aging.205798 (PMC11132012; doi:10.18632/aging.205798)
Supplement: Supplementary Table 1 [file aging-16-205798-s002.docx]

Supplementary Table 1. Information for 162 active ingredients of Xiaochaihu decoction.

| Drug | MolId | MolName | OB (%) | DL | DC |
| --- | --- | --- | --- | --- | --- |
| Banxia | MOL000358 | beta-sitosterol | 36.91 | 0.75 | 15 |
| Banxia | MOL000449 | Stigmasterol | 43.83 | 0.76 | 11 |
| Banxia | MOL000519 | coniferin | 31.11 | 0.32 | 10 |
| Banxia | MOL001755 | 24-Ethylcholest-4-en-3-one | 36.08 | 0.76 | 2 |
| Banxia | MOL002670 | Cavidine | 35.64 | 0.81 | 8 |
| Banxia | MOL002714 | baicalein | 33.52 | 0.21 | 28 |
| Banxia | MOL003578 | Cycloartenol | 38.69 | 0.78 | 1 |
| Banxia | MOL005030 | gondoic acid | 30.7 | 0.2 | 2 |
| Banxia | MOL006936 | 10,13-eicosadienoic | 39.99 | 0.2 | 2 |
| Banxia | MOL006957 | (3S,6S)-3-(benzyl)-6-(4-hydroxybenzyl) piperazine-2,5-quinone | 46.89 | 0.27 | 4 |
| Banxia | MOL006967 | beta-D-Ribofuranoside, xanthine-9 | 44.72 | 0.21 | 2 |
| Chaihu | MOL000098 | quercetin | 46.43 | 0.28 | 100 |
| Chaihu | MOL000354 | isorhamnetin | 49.6 | 0.31 | 20 |
| Chaihu | MOL000422 | kaempferol | 41.88 | 0.24 | 38 |
| Chaihu | MOL000449 | Stigmasterol | 43.83 | 0.76 | 11 |
| Chaihu | MOL000490 | petunidin | 30.05 | 0.31 | 8 |
| Chaihu | MOL001645 | Linoleyl acetate | 42.1 | 0.2 | 4 |
| Chaihu | MOL004598 | 3,5,6,7-tetramethoxy-2-(3,4,5-trimethoxyphenyl) chromone | 31.97 | 0.59 | 8 |
| Chaihu | MOL004609 | Areapillin | 48.96 | 0.41 | 11 |
| Chaihu | MOL004624 | Longikaurin A | 47.72 | 0.53 | 1 |
| Chaihu | MOL004653 | (+)-Anomalin | 46.06 | 0.66 | 2 |
| Chaihu | MOL004718 | α-spinasterol | 42.98 | 0.76 | 3 |
| Chaihu | MOL013187 | Cubebin | 57.13 | 0.64 | 4 |
| Dazao | MOL000096 | (-)-catechin | 49.68 | 0.24 | 8 |
| Dazao | MOL000098 | quercetin | 46.43 | 0.28 | 100 |
| Dazao | MOL000211 | Mairin | 55.38 | 0.78 | 1 |
| Dazao | MOL000358 | beta-sitosterol | 36.91 | 0.75 | 15 |
| Dazao | MOL000449 | Stigmasterol | 43.83 | 0.76 | 11 |
| Dazao | MOL000492 | (+)-catechin | 54.83 | 0.24 | 8 |
| Dazao | MOL000627 | Stepholidine | 33.11 | 0.54 | 9 |
| Dazao | MOL000787 | Fumarine | 59.26 | 0.83 | 9 |
| Dazao | MOL001454 | berberine | 36.86 | 0.78 | 11 |
| Dazao | MOL001522 | (S)-Coclaurine | 42.35 | 0.24 | 10 |
| Dazao | MOL002773 | beta-carotene | 37.18 | 0.58 | 21 |
| Dazao | MOL004350 | Ruvoside_qt | 36.12 | 0.76 | 2 |
| Dazao | MOL007213 | Nuciferin | 34.43 | 0.4 | 8 |
| Dazao | MOL012921 | stepharine | 31.55 | 0.33 | 3 |
| Dazao | MOL012946 | zizyphus saponin I_qt | 32.69 | 0.62 | 1 |
| Dazao | MOL012976 | coumestrol | 32.49 | 0.34 | 4 |
| Dazao | MOL012986 | Jujubasaponin V_qt | 36.99 | 0.63 | 1 |
| Dazao | MOL012992 | Mauritine D | 89.13 | 0.45 | 2 |
| Gancao | MOL000098 | quercetin | 46.43 | 0.28 | 100 |
| Gancao | MOL000211 | Mairin | 55.38 | 0.78 | 1 |
| Gancao | MOL000239 | Jaranol | 50.83 | 0.29 | 12 |
| Gancao | MOL000354 | isorhamnetin | 49.6 | 0.31 | 20 |
| Gancao | MOL000359 | sitosterol | 36.91 | 0.75 | 3 |
| Gancao | MOL000392 | formononetin | 69.67 | 0.21 | 23 |
| Gancao | MOL000417 | Calycosin | 47.75 | 0.24 | 19 |
| Gancao | MOL000422 | kaempferol | 41.88 | 0.24 | 38 |
| Gancao | MOL000497 | licochalcone a | 40.79 | 0.29 | 25 |
| Gancao | MOL000500 | Vestitol | 74.66 | 0.21 | 20 |
| Gancao | MOL001484 | Inermine | 75.18 | 0.54 | 7 |
| Gancao | MOL001792 | DFV | 32.76 | 0.18 | 7 |
| Gancao | MOL002311 | Glycyrol | 90.78 | 0.67 | 9 |
| Gancao | MOL002565 | Medicarpin | 49.22 | 0.34 | 16 |
| Gancao | MOL003656 | Lupiwighteone | 51.64 | 0.37 | 16 |
| Gancao | MOL003896 | 7-Methoxy-2-methyl isoflavone | 42.56 | 0.2 | 22 |
| Gancao | MOL004328 | naringenin | 59.29 | 0.21 | 22 |
| Gancao | MOL004805 | (2S)-2-[4-hydroxy-3-(3-methylbut-2-enyl) phenyl]-8,8-dimethyl-2,3-dihydropyrano[2,3-f] chromen-4-one | 31.79 | 0.72 | 9 |
| Gancao | MOL004806 | euchrenone | 30.29 | 0.57 | 5 |
| Gancao | MOL004808 | glyasperin B | 65.22 | 0.44 | 16 |
| Gancao | MOL004810 | glyasperin F | 75.84 | 0.54 | 14 |
| Gancao | MOL004811 | Glyasperin C | 45.56 | 0.4 | 17 |
| Gancao | MOL004814 | Isotrifoliol | 31.94 | 0.42 | 11 |
| Gancao | MOL004815 | (E)-1-(2,4-dihydroxyphenyl)-3-(2,2-dimethylchromen-6-yl) prop-2-en-1-one | 39.62 | 0.35 | 16 |
| Gancao | MOL004820 | kanzonols W | 50.48 | 0.52 | 17 |
| Gancao | MOL004824 | (2S)-6-(2,4-dihydroxyphenyl)-2-(2-hydroxypropan-2-yl)-4-methoxy-2,3-dihydrofuro[3,2-g] chromen-7-one | 60.25 | 0.63 | 16 |
| Gancao | MOL004827 | Semilicoisoflavone B | 48.78 | 0.55 | 12 |
| Gancao | MOL004828 | Glepidotin A | 44.72 | 0.35 | 18 |
| Gancao | MOL004829 | Glepidotin B | 64.46 | 0.34 | 8 |
| Gancao | MOL004833 | Phaseolinisoflavan | 32.01 | 0.45 | 16 |
| Gancao | MOL004835 | Glypallichalcone | 61.6 | 0.19 | 19 |
| Gancao | MOL004838 | 8-(6-hydroxy-2-benzofuranyl)-2,2-dimethyl-5-chromenol | 58.44 | 0.38 | 5 |
| Gancao | MOL004841 | Licochalcone B | 76.76 | 0.19 | 16 |
| Gancao | MOL004848 | licochalcone G | 49.25 | 0.32 | 14 |
| Gancao | MOL004849 | 3-(2,4-dihydroxyphenyl)-8-(1,1-dimethylprop-2-enyl)-7-hydroxy-5-methoxy-coumarin | 59.62 | 0.43 | 18 |
| Gancao | MOL004855 | Licoricone | 63.58 | 0.47 | 10 |
| Gancao | MOL004856 | Gancaonin A | 51.08 | 0.4 | 14 |
| Gancao | MOL004857 | Gancaonin B | 48.79 | 0.45 | 17 |
| Gancao | MOL004863 | 3-(3,4-dihydroxyphenyl)-5,7-dihydroxy-8-(3-methylbut-2-enyl) chromone | 66.37 | 0.41 | 14 |
| Gancao | MOL004864 | 5,7-dihydroxy-3-(4-methoxyphenyl)-8-(3-methylbut-2-enyl) chromone | 30.49 | 0.41 | 16 |
| Gancao | MOL004866 | 2-(3,4-dihydroxyphenyl)-5,7-dihydroxy-6-(3-methylbut-2-enyl) chromone | 44.15 | 0.41 | 12 |
| Gancao | MOL004879 | Glycyrin | 52.61 | 0.47 | 12 |
| Gancao | MOL004882 | Licocoumarone | 33.21 | 0.36 | 7 |
| Gancao | MOL004883 | Licoisoflavone | 41.61 | 0.42 | 15 |
| Gancao | MOL004884 | Licoisoflavone B | 38.93 | 0.55 | 12 |
| Gancao | MOL004885 | licoisoflavanone | 52.47 | 0.54 | 15 |
| Gancao | MOL004891 | shinpterocarpin | 80.3 | 0.73 | 16 |
| Gancao | MOL004898 | (E)-3-[3,4-dihydroxy-5-(3-methylbut-2-enyl) phenyl]-1-(2,4-dihydroxyphenyl) prop-2-en-1-one | 46.27 | 0.31 | 11 |
| Gancao | MOL004903 | liquiritin | 65.69 | 0.74 | 5 |
| Gancao | MOL004904 | licopyranocoumarin | 80.36 | 0.65 | 11 |
| Gancao | MOL004907 | Glyzaglabrin | 61.07 | 0.35 | 15 |
| Gancao | MOL004908 | Glabridin | 53.25 | 0.47 | 17 |
| Gancao | MOL004910 | Glabranin | 52.9 | 0.31 | 6 |
| Gancao | MOL004911 | Glabrene | 46.27 | 0.44 | 16 |
| Gancao | MOL004912 | Glabrone | 52.51 | 0.5 | 16 |
| Gancao | MOL004913 | 1,3-dihydroxy-9-methoxy-6-benzofurano[3,2-c] chromenone | 48.14 | 0.43 | 9 |
| Gancao | MOL004914 | 1,3-dihydroxy-8,9-dimethoxy-6-benzofurano[3,2-c] chromenone | 62.9 | 0.53 | 8 |
| Gancao | MOL004915 | Eurycarpin A | 43.28 | 0.37 | 15 |
| Gancao | MOL004924 | (-)-Medicocarpin | 40.99 | 0.95 | 1 |
| Gancao | MOL004935 | Sigmoidin-B | 34.88 | 0.41 | 5 |
| Gancao | MOL004941 | (2R)-7-hydroxy-2-(4-hydroxyphenyl) chroman-4-one | 71.12 | 0.18 | 8 |
| Gancao | MOL004945 | (2S)-7-hydroxy-2-(4-hydroxyphenyl)-8-(3-methylbut-2-enyl) chroman-4-one | 36.57 | 0.32 | 8 |
| Gancao | MOL004948 | Isoglycyrol | 44.7 | 0.84 | 6 |
| Gancao | MOL004949 | Isolicoflavonol | 45.17 | 0.42 | 12 |
| Gancao | MOL004957 | HMO | 38.37 | 0.21 | 19 |
| Gancao | MOL004959 | 1-Methoxyphaseollidin | 69.98 | 0.64 | 10 |
| Gancao | MOL004961 | Quercetin der. | 46.45 | 0.33 | 15 |
| Gancao | MOL004966 | 3'-Hydroxy-4'-O-Methylglabridin | 43.71 | 0.57 | 20 |
| Gancao | MOL004974 | 3'-Methoxyglabridin | 46.16 | 0.57 | 20 |
| Gancao | MOL004978 | 2-[(3R)-8,8-dimethyl-3,4-dihydro-2H-pyrano[6,5-f] chromen-3-yl]-5-methoxyphenol | 36.21 | 0.52 | 19 |
| Gancao | MOL004980 | Inflacoumarin A | 39.71 | 0.33 | 11 |
| Gancao | MOL004985 | icos-5-enoic acid | 30.7 | 0.2 | 1 |
| Gancao | MOL004988 | Kanzonol F | 32.47 | 0.89 | 6 |
| Gancao | MOL004989 | 6-prenylated eriodictyol | 39.22 | 0.41 | 6 |
| Gancao | MOL004990 | 7,2',4'-trihydroxy－5-methoxy-3－arylcoumarin | 83.71 | 0.27 | 13 |
| Gancao | MOL004991 | 7-Acetoxy-2-methylisoflavone | 38.92 | 0.26 | 17 |
| Gancao | MOL004993 | 8-prenylated eriodictyol | 53.79 | 0.4 | 6 |
| Gancao | MOL004996 | gadelaidic acid | 30.7 | 0.2 | 1 |
| Gancao | MOL005000 | Gancaonin G | 60.44 | 0.39 | 15 |
| Gancao | MOL005001 | Gancaonin H | 50.1 | 0.78 | 9 |
| Gancao | MOL005003 | Licoagrocarpin | 58.81 | 0.58 | 17 |
| Gancao | MOL005007 | Glyasperins M | 72.67 | 0.59 | 17 |
| Gancao | MOL005008 | Glycyrrhiza flavonol A | 41.28 | 0.6 | 13 |
| Gancao | MOL005012 | Licoagroisoflavone | 57.28 | 0.49 | 14 |
| Gancao | MOL005016 | Odoratin | 49.95 | 0.3 | 18 |
| Gancao | MOL005017 | Phaseol | 78.77 | 0.58 | 11 |
| Gancao | MOL005018 | Xambioona | 54.85 | 0.87 | 5 |
| Gancao | MOL005020 | dehydroglyasperins C | 53.82 | 0.37 | 14 |
| Huangqin | MOL000073 | ent-Epicatechin | 48.96 | 0.24 | 4 |
| Huangqin | MOL000173 | wogonin | 30.68 | 0.23 | 33 |
| Huangqin | MOL000228 | (2R)-7-hydroxy-5-methoxy-2-phenylchroman-4-one | 55.23 | 0.2 | 9 |
| Huangqin | MOL000358 | beta-sitosterol | 36.91 | 0.75 | 15 |
| Huangqin | MOL000359 | sitosterol | 36.91 | 0.75 | 3 |
| Huangqin | MOL000449 | Stigmasterol | 43.83 | 0.76 | 11 |
| Huangqin | MOL000525 | Norwogonin | 39.4 | 0.21 | 9 |
| Huangqin | MOL000552 | 5,2'-Dihydroxy-6,7,8-trimethoxyflavone | 31.71 | 0.35 | 13 |
| Huangqin | MOL001458 | coptisine | 30.67 | 0.86 | 6 |
| Huangqin | MOL001689 | acacetin | 34.97 | 0.24 | 23 |
| Huangqin | MOL002714 | baicalein | 33.52 | 0.21 | 28 |
| Huangqin | MOL002879 | Diop | 43.59 | 0.39 | 1 |
| Huangqin | MOL002897 | epiberberine | 43.09 | 0.78 | 7 |
| Huangqin | MOL002909 | 5,7,2,5-tetrahydroxy-8,6-dimethoxyflavone | 33.82 | 0.45 | 7 |
| Huangqin | MOL002910 | Carthamidin | 41.15 | 0.24 | 3 |
| Huangqin | MOL002913 | Dihydrobaicalin_qt | 40.04 | 0.21 | 3 |
| Huangqin | MOL002914 | Eriodyctiol (flavanone) | 41.35 | 0.24 | 5 |
| Huangqin | MOL002915 | Salvigenin | 49.07 | 0.33 | 11 |
| Huangqin | MOL002917 | 5,2',6'-Trihydroxy-7,8-dimethoxyflavone | 45.05 | 0.33 | 12 |
| Huangqin | MOL002925 | 5,7,2',6'-Tetrahydroxyflavone | 37.01 | 0.24 | 5 |
| Huangqin | MOL002927 | Skullcapflavone II | 69.51 | 0.44 | 12 |
| Huangqin | MOL002928 | oroxylin a | 41.37 | 0.23 | 19 |
| Huangqin | MOL002932 | Panicolin | 76.26 | 0.29 | 12 |
| Huangqin | MOL002933 | 5,7,4'-Trihydroxy-8-methoxyflavone | 36.56 | 0.27 | 15 |
| Huangqin | MOL002934 | NEOBAICALEIN | 104.34 | 0.44 | 14 |
| Huangqin | MOL002937 | DIHYDROOROXYLIN | 66.06 | 0.23 | 7 |
| Huangqin | MOL008206 | Moslosooflavone | 44.09 | 0.25 | 17 |
| Huangqin | MOL010415 | 11,13-Eicosadienoic acid, methyl ester | 39.28 | 0.23 | 1 |
| Huangqin | MOL012245 | 5,7,4'-trihydroxy-6-methoxyflavanone | 36.63 | 0.27 | 5 |
| Huangqin | MOL012246 | 5,7,4'-trihydroxy-8-methoxyflavanone | 74.24 | 0.26 | 4 |
| Huangqin | MOL012266 | rivularin | 37.94 | 0.37 | 15 |
| Renshen | MOL000358 | beta-sitosterol | 36.91 | 0.75 | 15 |
| Renshen | MOL000422 | kaempferol | 41.88 | 0.24 | 38 |
| Renshen | MOL000449 | Stigmasterol | 43.83 | 0.76 | 11 |
| Renshen | MOL000787 | Fumarine | 59.26 | 0.83 | 9 |
| Renshen | MOL002879 | Diop | 43.59 | 0.39 | 1 |
| Renshen | MOL003648 | Inermin | 65.83 | 0.54 | 9 |
| Renshen | MOL005308 | Aposiopolamine | 66.65 | 0.22 | 4 |
| Renshen | MOL005317 | Deoxyharringtonine | 39.27 | 0.81 | 2 |
| Renshen | MOL005318 | Dianthramine | 40.45 | 0.2 | 3 |
| Renshen | MOL005320 | arachidonate | 45.57 | 0.2 | 3 |
| Renshen | MOL005321 | Frutinone A | 65.9 | 0.34 | 8 |
| Renshen | MOL005344 | ginsenoside rh2 | 36.32 | 0.56 | 7 |
| Renshen | MOL005348 | Ginsenoside-Rh4_qt | 31.11 | 0.78 | 2 |
| Renshen | MOL005356 | Girinimbin | 61.22 | 0.31 | 5 |
| Renshen | MOL005376 | Panaxadiol | 33.09 | 0.79 | 1 |
| Renshen | MOL005384 | suchilactone | 57.52 | 0.56 | 8 |
| Renshen | MOL005399 | alexandrin_qt | 36.91 | 0.75 | 1 |
| Shengjiang | MOL000358 | beta-sitosterol | 36.91 | 0.75 | 15 |
| Shengjiang | MOL000449 | Stigmasterol | 43.83 | 0.76 | 11 |
| Shengjiang | MOL001771 | poriferast-5-en-3beta-ol | 36.91 | 0.75 | 2 |
| Shengjiang | MOL006129 | 6-methylgingediacetate2 | 48.73 | 0.32 | 3 |
