# Supplementary material for: Mechanistic exploration and experimental validation of the Xiaochaihu decoction for the treatment of breast cancer by network pharmacology
Source: Aging (Albany NY). 2024 May 13;16(9):7979–99. doi: 10.18632/aging.205798 (PMC11132012; doi:10.18632/aging.205798)
Supplement: Supplementary Table 2 [file aging-16-205798-s003.pdf]

Supplementary Table 2. Molecular docking binding energy.

Compound name	Gene	Binding energy (kcal/mol)
rutin	PI3K	-9.8
rutin	Akt	-10.6
quercetin	PI3K	-9.0
quercetin	Akt	-8.9
chrysin	PI3K	-8.9
chrysin	Akt	-8.5
naringenin	PI3K	-8.5
naringenin	Akt	-8.5
